# Perfect vs practical: utilizing hematology thin smears for the diagnosis of *Plasmodium* and *Babesia*

**DOI:** 10.1128/jcm.00644-25

**Published:** 2025-07-11

**Authors:** Neil Anderson

**Affiliations:** 1 Department of Pathology, Case Western Reserve School of Medicine 12304, Cleveland, Ohio, USA; Mayo Clinic Minnesota, Rochester, Minnesota, USA

## Abstract

In the manuscript entitled “Hematology Thin Smears Perform Equally to Parasitology Thick and Thin Blood Smears for the Diagnosis of *Plasmodium* and *Babesia* in a Low-Prevalence Setting,” the investigators demonstrate that traditional hematology smears (HS) review can have comparable performance to formal blood parasite smears (PS) review. The authors quote a 93.3% sensitivity in comparison to thick smears. This increases to 100% considering only new diagnoses. The implications are that HS can be utilized as a practical and rapid alternative to PS. For clinical laboratories that are limited to only HS capabilities, these findings suggest that they may be able to be less reliant on reference laboratory PS testing, allowing for more rapid diagnosis and management. Even in clinical laboratories that offer both tests, the findings suggest a reassessment of how both HS and PS fit into the diagnostic process for blood parasites. Familiarity with the pros and cons of both approaches allows them to be employed in a way that maximizes patient care impact.

## COMMENTARY

In the manuscript entitled “Hematology Thin Smears Perform Equally to Parasitology Thick and Thin Blood Smears for the Diagnosis of *Plasmodium* and *Babesia* in a Low-Prevalence Setting,” the authors challenge the dogma of what is required to perform an adequate blood parasite examination (1, 2). They directly compare two different methodologies for blood parasite detection: (i) the traditional gold standard of Giemsa stained thick and thin smears and (ii) Wright Giemsa staining performed in a general hematology laboratory. This latter methodology has traditionally been deemed less favorable for blood parasite examination, though it is the most common and readily accessible mechanism for blood smear analysis in the clinical laboratory (3, 4).

In this comparison, the authors made efforts to accurately model how both processes occur in real-world settings. Gold standard thick and thin smear testing (parasitology smears [PS]) was performed at a state public health laboratory by technologists with parasitology training and experience. Staining performed in the hematology laboratory (hematology smears [HS]) was performed 24/7 by technologists with more limited training. For HS staining, only thin smears were utilized, and the actual staining was performed utilizing the Wright Giemsa stain. This entire process effectively mimics the process by which most peripheral blood smears are prepared and read in the clinical laboratory.

Remarkably, the authors found little difference in performance when comparing approaches. The sensitivity of the HS approach was 93.3% when considering PS as the gold standard. When considering only newly diagnosed patients, this sensitivity increased to 100%. The implication of these findings is that HS alone may be an adequate screening approach in clinical settings for which PS is not readily available. Given the point of view of the investigators (state public health laboratory serving as a local parasitology reference laboratory), the message of the publication may be interpreted as a decreased need for clinical laboratories to be reliant on reference laboratories for blood parasite screening, which could conserve resources and allow for more rapid patient management. However, these findings could have broader implications even in settings in which both PS and HS are performed in the same clinical laboratory.

To understand these implications, one must understand how these processes currently coexist in most clinical laboratories today. Hematology staining and analysis often accompany general peripheral blood cell count testing, which is a test performed on nearly every patient receiving hospital care. As such, this testing is designed to be automated, rapid, and performed at all hours of the day. This is in direct contrast to traditional blood parasite staining. Traditional blood parasite staining requires manual preparation of both thick and thin smears, a staining process that often takes at least 45 minutes, and analysis by technologists expertly trained to read these specific slides (2). Given these differences, it is not surprising that while a provider may be able to have an HS performed rapidly any time of the day, PS is not a rapid test and is more often only offered during more limited timeframes. This is problematic in a critically ill patient for whom malaria or babesia is on the differential diagnosis. The dogma that these parasites should only be diagnosed utilizing traditional PS testing may lead to providers delaying patient management while awaiting the slower test. Furthermore, the perception that HS is an inappropriate approach for blood parasite diagnosis can undermine its utility when blood parasites are observed. If the perception is that “malaria is a parasitology test,” technologists performing HS analysis may lack the training to identify an infection. Even if they recognize an infection as abnormal and suspect a blood parasite, they may fail to recognize the importance of the finding and relegate reporting of the finding to their microbiology laboratory, delaying diagnosis.

So why do major laboratory guidelines state that blood parasite testing should ideally be performed utilizing Giemsa-stained thick and thin smears (2, 3)? There are several strong reasons for this endorsement.

1. The addition of thick smears enhances the sensitivity of the blood parasite exam. Mechanistically, this makes sense given the ability to screen a volume of blood in a properly prepared thick smear. This is supported by a variety of studies and academic sources (2, 3, 5). Compared to the thick smear, the sensitivity of the thin smear has been reported to be as low as 54.8% (6). The increased sensitivity of thick smears is of particular importance when considering patients with low parasitemia (7).
2. Giemsa staining is better suited for blood parasite detection compared to Wright Giemsa staining. The Giemsa stain takes longer to perform, though it results in a more intense highlighting of nuclear, chromatin, bacterial, and parasitic detail (8). The Wright Giemsa stain is a combination of two stains, the Wright and Giemsa stains. The staining process is faster, and the addition of the Wright stain helps better highlight red blood cells. The tradeoff is that although parasites can be visualized by the Wright Giemsa stain, certain structures (i.e., Schuffner’s stippling or Maurer’s clefts) will not be visible (5).

Given these differences, it may be surprising that the investigators demonstrated nearly comparable performance between HS and PS testing. However, the findings seem more plausible when one considers the setting and way in which HS was utilized. Testing was performed in a hospital setting in a low-prevalence geographic region. Most patients presenting with an infection were likely newly diagnosed and untreated and, therefore, had moderate parasitemias. It has been well established that the sensitivity of the thin smear increases with a high percentage of parasitemias (7). As such, in this population, the added sensitivity of a thick smear was likely of less utility. This is supported by the authors’ findings that HS had 100% sensitivity in newly diagnosed patients.

It is also worth noting that the Wright Giemsa stain was likely adequate for the purpose for which it was utilized in this study. HS smears were not utilized for definitive species-level identification. Rather, they were utilized to screen for the presence of blood parasites. Definitive identification was reserved for the Giemsa examination, which is more appropriate given the better morphologic detail observed by this technique. Even if a definitive identification cannot be rendered immediately, generally knowing a patient has a blood parasite infection allows providers to make timely decisions about patient care while awaiting definitive results.

This study is important because it highlights that the Wright Giemsa smear can play an important role in the diagnosis of blood parasites. Clinical laboratories should consider this as they evaluate the training of technologists and diagnostic workflows. The identification and subsequent reporting of potential blood parasites should be part of the training of all technologists who perform the Wright Giemsa smear examination. While definitive identification may be challenging with the Wright Giemsa or beyond the training of all technologists performing HS, having mechanisms to report the possible presence of blood parasites may allow for more timely patient management.

Furthermore, if a formal blood parasite examination is ordered and a delay is anticipated, efforts can be made to have parallel Wright Giemsa staining ordered as well. While providers often do order these tests in parallel, it is also important for them to understand that the HS examination can be utilized as a rapid screen while awaiting PS examination (if the laboratory is formally evaluating HS for blood parasites and reporting the findings). Complicating this is the fact that reporting of complete blood cell results is often performed utilizing automated technology. The performance of these instruments for the detection of blood parasites is variable and of limited study. For one commonly utilized flow cytometry-based instrument, the Sysmex hematology analyzer, the sensitivity has been reported to range from 46% to 100%, depending on the study (9). Yet another automated analyzer, relying on digital image analysis from prepared smears, yielded an overall sensitivity of 23.5% (10). In many laboratories, peripheral smears are only performed when abnormalities are detected by these automated instruments or when a peripheral smear review is manually ordered. As such, for providers to use HS as a rapid screen, there needs to be reporting clarity regarding whether a manual review of the stain was performed. Making a manual review option accessible on all shifts will better allow Wright Giemsa staining to serve as a rapid blood parasite screen.

The results presented in this study do not suggest a lack of a role for PS examination. Species-level identification is important for the treatment of malaria and is best performed utilizing the PS examination. Furthermore, the performance of HS seen in this study may not be reproducible across all locations. The manual review of slides is heavily dependent on the training and experience of the reviewer. This was only a limited sampling (*n* = 45 positives) in a single institution. Additional, multicenter studies would be needed to capture the true variability of HS analysis.

It is also worth noting that there are other rapid and practical diagnostics that can be utilized for malaria diagnosis. Rapid malaria antigen tests are relatively easy to perform and, in some studies, have been shown to have superior diagnostic performance in comparison to traditional PS examination (11). However, these tests may not be as readily available in every setting as HS analysis. Furthermore, HS analysis is often ordered for other reasons, while rapid malaria antigen testing requires a provider to identify malaria as a differential diagnosis up front. This may not consistently happen in a non-endemic area.

What can be concluded from this study, though, is that, in at least some settings, HS can be viewed as an adequate and practical approach to blood parasite examination when gold standard PS examination is not readily available. Guidelines and protocols that singularly endorse thick and thin smear Giemsa stain examination for blood parasite examination should consider the role traditional hematology staining can play in diagnosis. We commonly accept more practical yet less accurate methodologies of testing for other pathogens (i.e., antigen testing for SARS-CoV-2). Provided the strengths and limitations of the different approaches are understood, there is not a good reason to consider blood parasite testing differently. Perhaps Voltaire said it best: too often we let the best be the enemy of the good.
